# Monckeberg Medial Calcific Sclerosis of the Temporal Artery Masquerading as Giant Cell Arteritis: Case Reports and Literature Review

**DOI:** 10.7759/cureus.9210

**Published:** 2020-07-15

**Authors:** Francisco J Cuevas Castillo, Sunam Sujanani, Vishaka K Chetram, Mohanad Elfishawi, Adriana Abrudescu

**Affiliations:** 1 Medicine, Icahn School of Medicine, Mount Sinai/Queens Hospital Center, New York, USA; 2 Internal Medicine, NYC Health + Hospitals/Queens, New York, USA; 3 Internal Medicine, MedStar Washington Hospital Center, Washington, D.C., USA; 4 Rheumatology, Icahn School of Medicine, Mount Sinai/Queens Hospital Center, New York, USA

**Keywords:** monckeberg medial calcific sclerosis, giant cell arteritis, temporal artery biopsy

## Abstract

Monckeberg medial calcific sclerosis (MCS) is an infrequent finding in the temporal artery and can clinically present almost indistinguishably from giant cell arteritis (GCA). To our knowledge, there have been only two case reports of suspected GCA found to be MCS only after a temporal artery biopsy (TAB).

Herein, we present two cases. The first case is a 69-year-old female with hypertension, type-2 diabetes mellitus, and chronic headaches who presented with left temporal headaches and scalp tenderness. She had a prominently dilated, tortuous, and tender left temporal artery. Initial labs showed a leukocyte count of 11.1x10^3^/L, erythrocyte sedimentation rate (ESR) of 29 mm/hr, and C-reactive protein (CRP) of 5.8 mg/L. The patient was started on prednisone 60 mg for presumptive GCA. Left TAB was negative for inflammatory changes, with findings consistent with MCS. Steroids were discontinued, and symptoms resolved. The second case is a 67-year-old male with hypertension, asthma, hyperlipidemia, status-post left eye cataract phacoemulsification, with intraocular lens insertion one-month prior, who presented with left eye blurriness in the inferior visual field and intermittent headache for 15 days. Left ophthalmoscopy showed retinal pallor and edema. Initial labs revealed ESR of 25 mm/hr, CRP of 11.2 mg/L, leukocyte count of 13.01x10^3^/L. The patient was given solumedrol 120 mg once and prednisone 70 mg daily for presumptive GCA. Left TAB was negative for GCA but reported damaged elastic fibers by calcification consistent with MCS. Partial visual blurriness remained, and steroids were discontinued.

This report accentuates the importance of MCS as a temporal GCA simulator. Physicians should be aware that TAB potentially changes management and may help surface underlying conditions.

## Introduction

Giant cell arteritis (GCA) is a chronic, idiopathic, granulomatous vasculitis of the medium and large arteries [1]. It is mostly found in people above 50 years of age and usually involves the branches of the external and internal carotid arteries, predominantly the temporal artery, leading to the classical symptoms of headache, scalp tenderness, jaw claudication, and visual impairment.

Monckeberg medial calcific sclerosis (MCS) is described as calcification of the tunica media and/or internal elastic lamina in small and medium-sized arteries, commonly, but not exclusively, associated with aging and numerous comorbidities ranging from type 2 diabetes mellitus and renal dysfunction to hormone disorders and vitamin deficiencies [2]. This form of arteriosclerosis is most often detected in the muscular arteries of the extremities and, sporadically, the visceral ones.

Clinically, GCA of the temporal artery is a medical emergency and should be treated punctually since it can progress to permanent vision loss [3], while MCS is usually asymptomatic, often discovered incidentally on radiographs or ultrasounds of the pelvis or lower limbs, and identified as linear radiopaque lesions with an ankle-brachial index greater than 1.1 [1].

To date, few cases of MCS involving the temporal artery have been documented. In this case report, we describe two cases of MCS of the temporal artery presenting as suspected GCA.

## Case presentation

Case 1

A 69-year-old female, with a past medical history of hypertension, type 2 diabetes mellitus, and chronic headaches, was sent to the emergency room by her private ophthalmologist for the evaluation of worsening left temporal headaches and left scalp tenderness.

The patient reported worsening bitemporal headaches for the last six months (left worse than right), described as throbbing in nature and associated with photophobia, phonophobia, and nausea. She also noted left scalp tenderness, jaw claudication with chewing, and bilateral shoulder pain. She denied any visual changes, neck stiffness, tinnitus, focal neurological deficit, fever, weight loss, or rash.

On physical examination, a prominently dilated and tortuous left temporal artery was noted, which was tender to palpation. Initial labs were notable for a leukocyte count of 11.1 x103/L, hemoglobin of 12 g/dL, platelet count of 199 x103/L, creatinine of 1.14 mg/dL, erythrocyte sedimentation rate (ESR) of 29 mm/hr and C-reactive protein (CRP) of 5.8 mg/L. Non-contrast CT head was negative for pathology.

**Figure 1:**
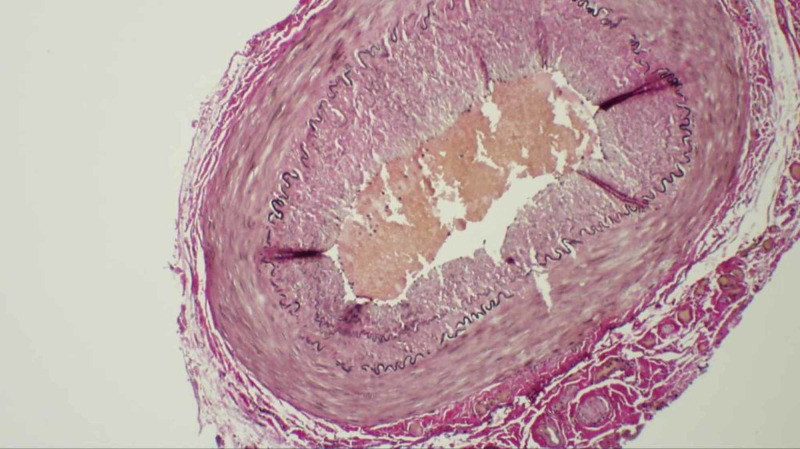
Temporal artery biopsy showing calcification of tunica media

Based on clinical findings and the presumed diagnosis of GCA, the patient was started on prednisone 60 mg daily. Left temporal artery biopsy (TAB) was performed two days after steroid therapy was initiated. The pathology report was negative for any inflammatory changes and demonstrated findings consistent with MCS (Figure 1). Symptoms resolved, and steroids were discontinued after one week. The patient remained asymptomatic at the three-week follow-up visit and was diagnosed with temporomandibular joint dysfunction.

Case 2

A 67-year-old male with a past medical history of hypertension, asthma, hyperlipidemia, status-post left eye phacoemulsification cataract procedure, with the insertion of an intraocular lens in the left eye one month prior, presented to the emergency room with complaints of blurry vision in the inferior visual field of the left eye and intermittent headache for 15 days.

Upon presentation, the patient reported that, initially, his vision was clear postoperative for one week but became blurry in the inferior field of vision of the left eye with gradual deterioration. The patient denied eye pain, redness, vision loss, neck pain, fever, ataxia, diplopia, or jaw claudication.

Evaluation of the left eye showed retinal pallor and edema. No prominent temporal artery or scalp tenderness was appreciated, and the rest of the physical examination was unremarkable. Laboratory results upon presentation revealed an ESR of 25 mm/hr, CRP of 11.2 mg/L, white blood cell (WBC) count of 13.01 x103/L, hemoglobin of 13.3 g/dL, hematocrit of 40.8%, neutrophil count of 78.7%, and lymphocyte count of 13.2%. Non-contrast computed tomography (CT) head and the carotid duplex was negative for pathology.

**Figure 2:**
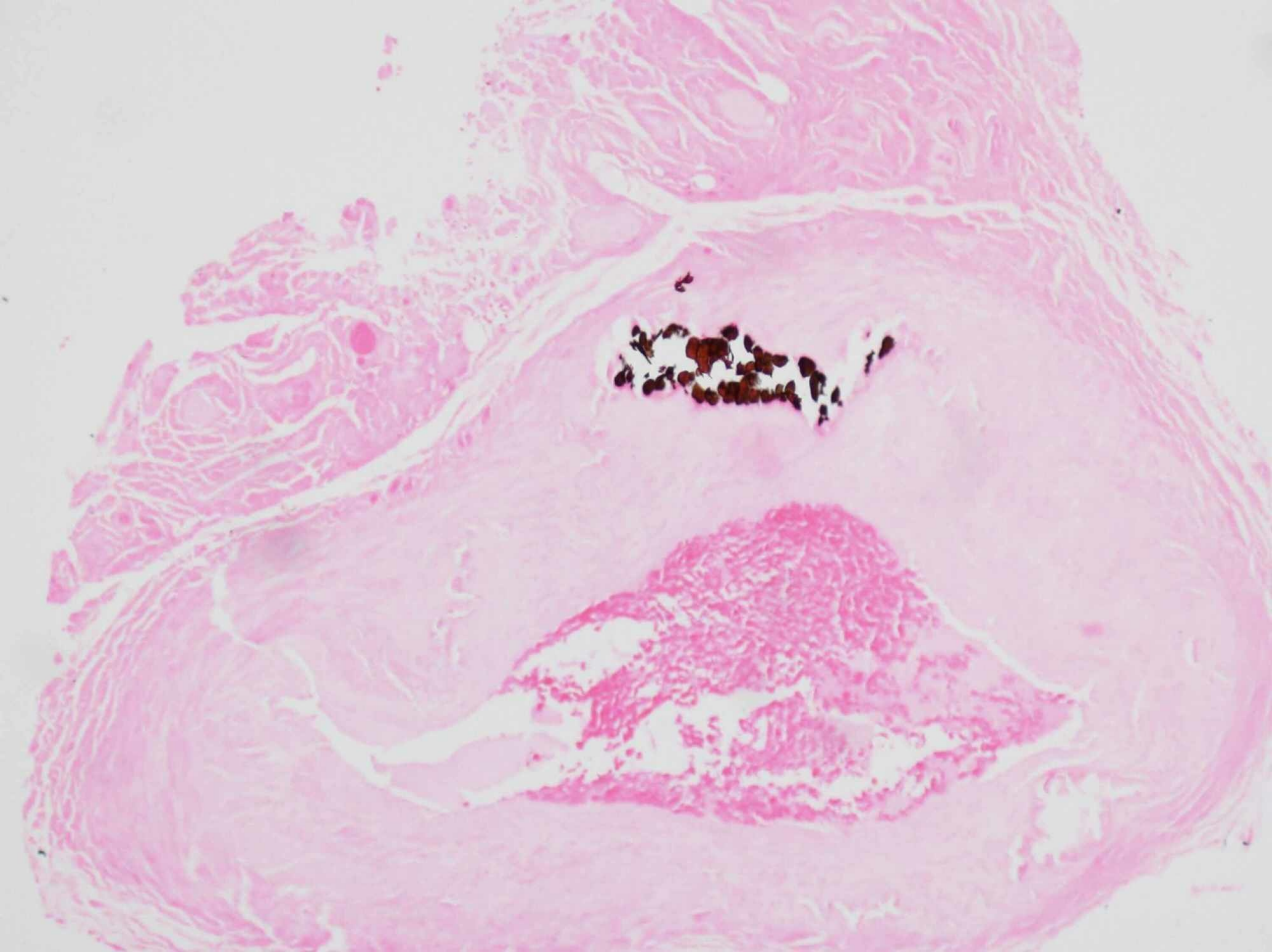
Temporal artery biopsy Calcium stain demonstrating Monckeberg medial calcific sclerosis

**Figure 3:**
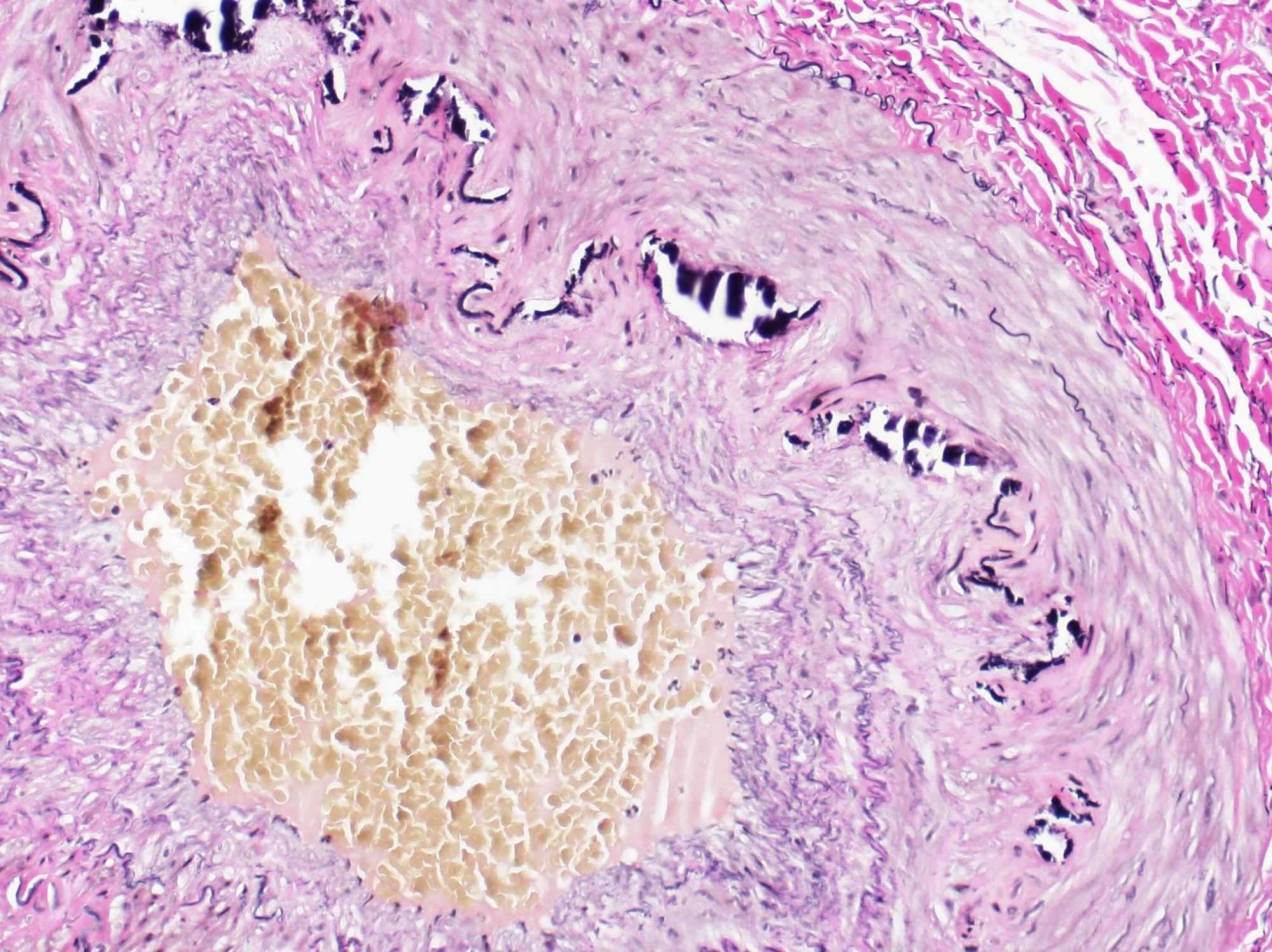
Temporal artery biopsy Elastic fiber stain showing disruption secondary to calcification

Based on clinical findings and concern for potential GCA, solumedrol 120 mg was given in the emergency room and the patient was started on prednisone 70 mg daily. Left temporal artery biopsy was performed six days after steroid therapy was initiated. The pathology report was negative for GCA and demonstrated damage to elastic fibers by calcification consistent with MCS (Figures 2-3). Prednisone was discontinued and, due to persistently blurry vision, the patient was diagnosed with nonarteritic anterior ischemic optic neuropathy on his three-week follow-up visit.

## Discussion

This report describes two cases of temporal artery MCS simulating GCA. To the best of our knowledge, only two other cases were published with similar presentations and biopsy reports (Table 1).

**Table 1:** Summary of reported cases in the literature ESR: erythrocyte sedimentation rate; CRP: C-reactive protein; GERD: gastroesophageal reflux disease

Case report	Age/sex	Clinical presentation	Inflammatory markers (ESR in mm/hour, CRP in mg/L)	Comorbidities	Biopsy report	Outcome
Present case 1 (2020)	69 / F	Chief complaint: Bitemporal headache x 6 months. Pertinent findings: Photophobia, phonophobia, nausea, jaw claudication, left scalp tenderness, dilated, tortuous left temporal artery	ESR: 29, CRP: 5.8	Hypertension, type 2 diabetes, headaches	Focal internal elastic lamina destruction, mild atherosclerosis with intimal hyperplasia, no inflammation	Symptoms resolved
Present case 2 (2020)	67 / M	Chief complaint: Blurry vision in left eye for 3 days, intermittent headache for 15 days. Pertinent findings: Dizziness	ESR: 25, CRP: 11.2	Hypertension, asthma, hyperlipidemia, GERD	Partial damage of elastic fibers by calcification	Blurry vision in the lower visual field of the left eye persists
Phelps PO et al. (2015) [3]	Not reported	Chief complaint: Acute vision loss. Pertinent findings: Hard artery to palpation	ESR: normal, CRP: normal	Not reported	Mineralization of internal elastic lamina	Irreversible vision loss
Belliveau et al. (2013) [4]	85 / F	Chief complaint: Left temporal tenderness for 2 weeks, intermittent headache for 15 days. Pertinent findings: Left-sided hearing loss, jaw claudication	ESR: 23, CPR: 1.0	Osteoporosis, hypothyroidism, coronary artery disease, osteoarthritis, lung neoplasm (active)	Focal ossification of media and internal elastic lamina, visible osteoclast-type giant cells, lumen was patent	Not specified

Out of the four cases, three of them were above 50 years of age and experienced headaches; two of them manifested tenderness in the temporal area; and one of the cases presented with acute vision loss and temporal artery hardening. Although these four cases portrayed normal ESR, the concurrence of temporal arteritis and normal ESR has been described in the literature, where a meta-analysis of 114 studies suggested that physical findings characteristic of this vasculitis yielded more value for positive diagnosis than the significance of an elevated ESR for ruling out GCA [5-6].

Currently, in the setting of signs and symptoms suggestive of GCA [7], prompt evaluation of the temporal artery via color Doppler ultrasound (CDUS) or temporal artery biopsy is recommended. CDUS of the head, neck, and upper extremities has been suggested as a diagnostic aid for GCA due to its non-invasiveness and the specificity reported of the “halo sign” (which represents mural edema) [8], although results are operator-dependent in nature and the procedure has not yet been standardized. In our case reports, CDUS was not performed due to a lack of extensive experience.

TAB remains the gold standard for the diagnosis of temporal arteritis, in which case, intimal thickening with luminal stenosis, mononuclear inflammatory cell infiltrate with medial invasion, necrosis, and medial giant cell formation are hallmark features [5]. False-negative TAB in suspected GCA can occur, likely due to the sampling of skip lesions. Nonetheless, in a study of TAB results performed on patients with suspected GCA, 6% of specimens showed MCS associated with disorganization around the calcification and disruption of the internal elastic lamina, findings that should not be erroneously interpreted as sequelae of previous arterial inflammation [9].

Initially defined in 1903 in the arteries of the extremities [10], medial calcification has been observed histologically in the ascending aorta, medium-sized renal arteries, and, less commonly, small arteries, including the coronaries, the temporals, and others [2,11-12], with an approximate prevalence of 27% [13] in patients with end-stage renal disease, 17% in newly diagnosed diabetics [14], and 41% in those with more advanced disease [15]. In the microcirculation, this leads to altered hemodynamics, loss of autoregulation, and impaired compensatory remodeling [16].

As vascular compromise progresses, there is a loss of arterial wall elasticity, progressively deteriorating anterior arterial flow, thrombus formation, and decreased organ perfusion [2]. It is important to distinguish MCS from other forms of vascular calcification, such as calciphylaxis, since the former is an innocuous form where calcium deposits assume a quiescent pattern of deposition without true endoluminal calcification or vascular compromise, while the latter describes a sudden precipitous deposition of calcium in soft tissue and is associated with endoluminal calcification and vascular thrombotic occlusion [17].

The physiologic underpinnings, clinical consequences, and therapies for MCS remain elusive, including whether the condition is localized or systemic. Nonetheless, medial calcification has been recognized as an independent risk factor for cardiovascular events and all-cause mortality [13-14,18].

## Conclusions

To conclude, this article accentuates the significance of MCS as a simulator of temporal GCA. Primary physicians, rheumatologists, ophthalmologists, and pathologists should be aware of this diagnosis since its identification on TAB greatly changes the management of patients with suspected temporal arteritis and may be a sign of potential underlying conditions such as diabetes mellitus, chronic kidney disease, and cardiovascular risk factors.
